# SqueezeMeta, A Highly Portable, Fully Automatic Metagenomic Analysis Pipeline

**DOI:** 10.3389/fmicb.2018.03349

**Published:** 2019-01-24

**Authors:** Javier Tamames, Fernando Puente-Sánchez

**Affiliations:** Department of Systems Biology, Spanish Center for Biotechnology, CSIC, Madrid, Spain

**Keywords:** binning, metagenomics, MinION, RNAseq, software

## Abstract

The improvement of sequencing technologies has facilitated generalization of metagenomic sequencing, which has become a standard procedure for analyzing the structure and functionality of microbiomes. Bioinformatic analysis of sequencing results poses a challenge because it involves many different complex steps. SqueezeMeta is a fully automatic pipeline for metagenomics/metatranscriptomics, covering all steps of the analysis. SqueezeMeta includes multi-metagenome support that enables co-assembly of related metagenomes and retrieval of individual genomes via binning procedures. SqueezeMeta features several unique characteristics: co-assembly procedure or co-assembly of unlimited number of metagenomes via merging of individual assembled metagenomes, both with read mapping for estimation of the abundances of genes in each metagenome. It also includes binning and bin checking for retrieving individual genomes. Internal checks for the assembly and binning steps provide information about the consistency of contigs and bins. Moreover, results are stored in a MySQL database, where they can be easily exported and shared, and can be inspected anywhere using a flexible web interface that allows simple creation of complex queries. We illustrate the potential of SqueezeMeta by analyzing 32 gut metagenomes in a fully automatic way, enabling retrieval of several million genes and several hundreds of genomic bins. One of the motivations in the development of SqueezeMeta was producing a software capable of running in small desktop computers and thus amenable to all users and settings. We were also able to co-assemble two of these metagenomes and complete the full analysis in less than one day using a simple laptop computer. This reveals the capacity of SqueezeMeta to run without high-performance computing infrastructure and in absence of any network connectivity. It is therefore adequate for *in situ*, real time analysis of metagenomes produced by nanopore sequencing. SqueezeMeta can be downloaded from https://github.com/jtamames/SqueezeMeta.

## Introduction

The improvement of sequencing technologies has permitted the generalization of metagenomic sequencing, which has become standard procedure for analyzing the structure and functionality of microbiomes. Many novel bioinformatic tools and approaches have been developed to deal with the vast numbers of short read sequences produced by a metagenomic experiment. Aside from the simply overwhelming amount of data, a metagenomic analysis is a complex task comprising several non-standardized steps, involving different software tools whose results are often not directly compatible.

Lately, the development of highly portable sequencers, especially those based on nanopore technologies (Deamer et al., 2016), has facilitated *in situ* sequencing in scenarios where the need to obtain quick results is paramount, for instance clinical scenarios of disease control or epidemics (Quick et al., 2015, 2016). Metagenomic sequencing has also been performed *in situ*, for instance in oceanographic expeditions in the Antarctic ice (Lim et al., 2014; Johnson et al., 2017), illustrating the growing capability of producing sequences right away in sampling campaigns. This will enable informed planning of upcoming sampling experiments according to the results found in previous days. We foresee that this kind of application will be increasingly used in the near future. Therefore, bioinformatic analysis should be performed in a very short time span (hours), and be amenable to lightweight computing infrastructure.

A standard metagenomic pipeline involves read curation, assembly, gene prediction, and functional and taxonomic annotation of the resulting genes. Several pipelines have been created to automate most of these analyses (Li, 2009; Arumugam et al., 2010; Glass and Meyer, 2011; Abubucker et al., 2012; Eren et al., 2015; Kim et al., 2016). However, they differ in terms of capacities and approaches. One of the most important differences is whether or not the assembly step is needed. Some platforms skip assembly and, consequently, gene prediction and rely instead on direct annotation of the raw reads. Nevertheless, there are several drawbacks of working with raw reads: since this is based on homology searches for millions of sequences against huge reference databases, it usually requires very large CPU usage. Especially for taxonomic assignment, the reference database must be as complete as possible to minimize errors (Pignatelli et al., 2008). Furthermore, sequences are often too short to produce accurate assignments (Wommack et al., 2008; Carr and Borenstein, 2014).

Assembly, however, is advisable because it can recover larger fragments of genomes, often comprising many genes. Having the complete sequence of a gene and its context makes its functional and taxonomic assignment much easier and more reliable. The drawback of assembly is the formation of chimeras because of misassembling parts of different genomes, and the inability to assemble some of the reads, especially the ones from low-abundance species. The fraction of non-assembled reads depends on several factors, especially sequencing depth and microbiome diversity, but it is usually low (often below 20%). Recently, some tools have been developed to reassemble the portion of reads not assembled in the first instance, increasing the performance of this step (Hitch and Creevey, 2018). Co-assembling related metagenomes can also alleviate this problem significantly, as we will illustrate in the results section.

Assembly is also advisable because it facilitates the recovery of quasi-complete genomes via binning methods. The retrieval of genomes is a major step forward in the study of a microbiome, since it enables linking organisms and functions, thereby contributing to a much more accurate ecologic description of the community’s functioning. It is possible, for instance, to determine the key members of the microbiome (involved in particularly important functions), to infer potential interactions between members (for instance, looking for metabolic complementation), and to advance in the understanding of the effect of ecologic perturbations.

The best strategy for binning is co-assembly of related metagenomes. By comparing the abundance and composition of the contigs in different samples, it is possible to determine which contigs belong to the same organism: these contigs have similar oligonucleotide composition, similar abundances in individual samples, and a co-varying pattern between different samples. In this way, it is possible to retrieve tens or hundreds of genomic bins with different levels of completion that can be used as the starting point for a more in-depth analysis of the microbiome’s functioning.

SqueezeMeta is a fully automatic pipeline for metagenomics/metatranscriptomics, covering all steps of the analysis. It includes multi-metagenome support allowing co-assembly of related metagenomes and the retrieval of individual genomes via binning procedures.

A comparison of the capabilities of SqueezeMeta and other pipelines is shown in Table 1. Most current pipelines do not include support for co-assembling and binning, while some permit importing external binning results to display the associated information.

**Table 1:** Features of different metagenomic analysis pipelines, in comparison to SqueezeMeta.

	MG-Rast (Meyer et al., 2008)	Anvio (Eren et al., 2015)	Smash community (Arumugam et al., 2010)	Humann (Abubucker et al., 2012)	fmap (Kim et al., 2016)	MetaWrap (Uritskiy et al., 2018)	Samsa2 (Westreich et al., 2018)	IMP (Narayanasamy et al., 2016)	Squeeze Meta
Assembly	No	No	Yes	No	No	Yes	No	Yes	Yes
Data source	Reads or contigs	Contigs	Contigs	Reads	Reads or contigs	Contigs	Reads (RNA)	Reads	Reads
Gene prediction	Yes	Yes	Yes	No	No	No	No	Yes	Yes
Function assignment	Yes	Yes	Yes	Yes	Yes	No	Yes	Yes	Yes
RNA assignment	Yes	Yes	No	No	No	No	Yes	Yes	Yes
Taxonomic assignment	Yes	Yes	Yes	Yes	Yes	Yes	Yes	Yes	Yes
Gene abundances	Yes	Yes	No	Yes	Yes	No	Yes	Yes	Yes
Metagomic comparison	Yes	Yes	Yes	Yes	Yes	No	Yes	Yes	Yes
Co-assembly	No	No	No	No	No	Yes	No	Yes	Yes
Binning	No	Support	No	No	No	Yes	No	Yes	Yes
Bin validation	No	Yes	No	No	No	No	No	No	Yes
Local Installation	No	Yes	Yes	Yes	Yes	Yes	Yes	Yes	Yes


SqueezeMeta offers several advanced characteristics that make it different to existing pipelines, for instance:

1. Co-assembly procedure coupled with read mapping for the estimation of the abundances of individual genes in each metagenome.
2. An alternative co-assembly approach enabling the processing of an unlimited number of metagenomes via merging of individual metagenomes.
3. Support for nanopore long reads.
4. Binning and bin checking for retrieving individual genomes.
5. Internal checks for the taxonomic annotation of contigs and bins.
6. Metatranscriptomic support via mapping of cDNA reads against reference metagenomes, or via co-assembly of metagenomes and metatranscriptomes.
7. Inclusion of MySQL database for storing results, where they can be easily exported and shared and inspected anywhere using a web interface.

We have designed SqueezeMeta to be able to run in scarce computer resources, as expected for *in situ* metagenomic sequencing experiments. By adequately setting all the pipeline’s components, we were able to fully analyze completely individual metagenomes and even co-assemble related metagenomes using a desktop computer with only 16 GB RAM. The fully automatic nature of our system, not requiring any technical or bioinformatic knowledge, also makes it very easy to use. It is also completely independent of the availability of any Internet connection.

SqueezeMeta can be downloaded from https://github.com/jtamames/SqueezeMeta.

## Materials and Methods

**FIGURE 1:**
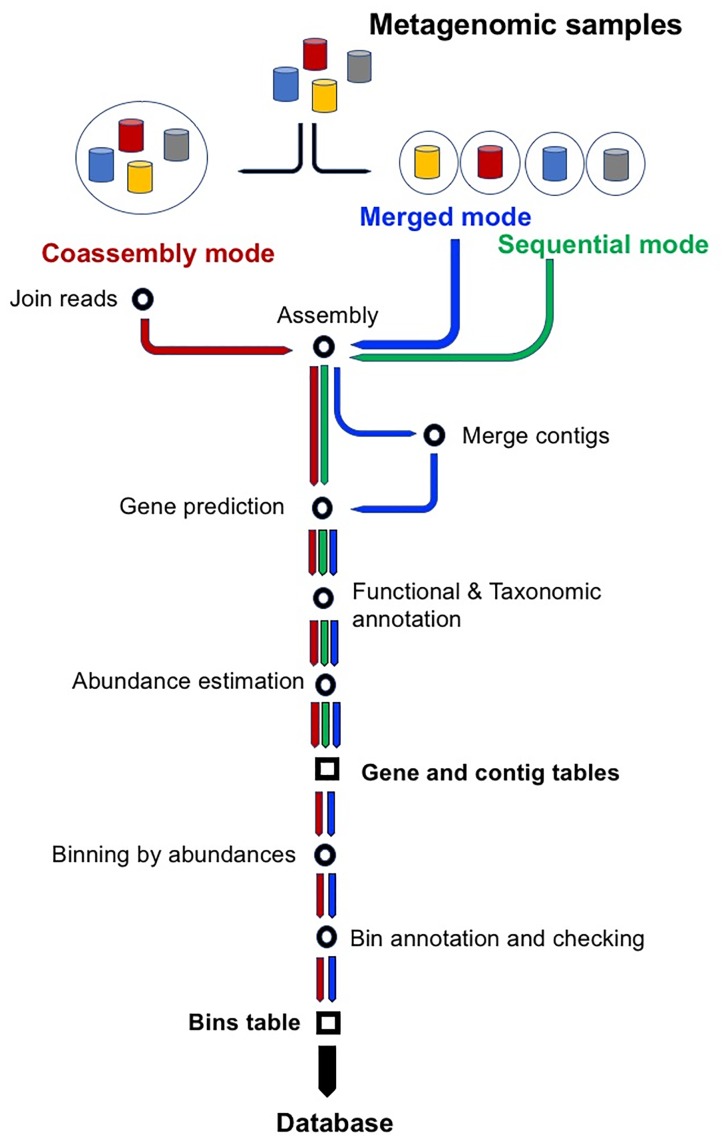
Workflow of the three modes of operation of SqueezeMeta: sequential, co-assembly and merged. Starting from metagenomic samples, green, blue and red arrows indicate main steps in sequential, merged and co-assembly modes. All modes create two of the three main results tables: ORF and contig tables. Co-assembly and merged modes also apply binning and, therefore, they also create the bin table.

SqueezeMeta is aimed to perform the analysis of several metagenomes in a single run. It can be run in three different modes (for a schematic workflow for the three modes, see Figure 1). These are:

1. Sequential mode: all metagenomes are treated individually and analyzed sequentially. This mode does not include binning, since each metagenome is treated independently.
2. Co-assembly mode: reads from all samples are pooled and a single assembly is performed. Reads from individual samples are then mapped back to the co-assembly, which enables obtaining the coverage of contigs and individual genes in these contigs. Based on these abundances, subsequent binning methods allow classifying contigs in genomic bins.
3. Merged mode: co-assembly is a very intensive process that requires plenty of computational resources, especially RAM. If the number of samples is high, requirements can easily exceed the capabilities of the computing infrastructure. SqueezeMeta’s merged mode permit co-assembly of a large number of samples, using a procedure similar to the one used in the analysis of TARA Oceans metagenomes (Tully et al., 2018). Samples are first assembled individually. The resulting sets of contigs are merged by combining contigs with ≥99% semi-global identity, using CD-HIT (Fu et al., 2012). Then the remaining contigs are re-assembled using Minimus2 (Treangen et al., 2011) with parameters -D OVERLAP = 100 MINID = 95, to look for overlapping contigs coming from pieces of the same genome in different samples. The merging produces a single set of contigs, and the analysis proceeds as in the co-assembly mode.

SqueezeMeta uses a combination of custom scripts and external software packages for the different steps of the analysis. A more detailed description of these steps follows:

### Data Preparation

A SqueezeMeta run only requires a configuration file indicating the metagenomic samples and the location of their corresponding sequence files. The program creates the appropriate directories and prepares the data for further steps.

### Trimming and Filtering

SqueezeMeta uses Trimmomatic for adapter removal, trimming and filtering by quality, according to the parameters set by the user (Bolger et al., 2014).

### Assembly

When assembling large metagenomic datasets, computing resources, especially memory usage, are critical. SqueezeMeta uses Megahit (Li et al., 2015) as its reference assembler, since we find it has an optimal balance between performance and memory usage. SPAdes (Bankevich et al., 2012) is also supported. For assembly of the long, error-prone MinION reads, we use Canu (Koren et al., 2017). The user can select any of these assemblers. In the merged mode, each metagenome will be assembled separately and the resulting contigs will be merged and joined as outlined above. Either way, the resulting set of contigs is filtered by length using prinseq (Schmieder and Edwards, 2011), to discard short contigs if required.

### Gene and rRNA Prediction

This step uses the Prodigal gene prediction software (Hyatt et al., 2010) to perform a gene prediction on the contigs, retrieving the corresponding amino acid sequences, and looks for rRNAs using barrnap (Seemann, 2014). The resulting 16S rRNA sequences are classified using the RDP classifier (Wang et al., 2007).

### Homology Searching

SqueezeMeta uses the Diamond software (Buchfink et al., 2015) for comparison of gene sequences against several taxonomic and functional databases, because of its optimal computation speed while maintaining sensitivity. Currently, three different Diamond runs are performed: against the GenBank nr database for taxonomic assignment, against the eggNOG database (Huerta-Cepas et al., 2016) for COG/NOG annotation, and against the latest publicly available version of KEGG database (Kanehisa and Goto, 2000) for KEGG ID annotation. SqueezeMeta also classifies genes against the PFAM database (Finn et al., 2014), using HMMER3 (Eddy, 2009). These databases are installed locally and updated at the user’s request.

### Taxonomic Assignment of Genes

Custom scripts are used for this step of the analysis. For taxonomic assignment, SqueezeMeta implements a fast LCA algorithm that looks for the last common ancestor of the hits for each query gene using the results of the Diamond search against GenBank nr database (the most complete reference database available). For each query sequence, we select a range of hits having at least 80% of the bit-score of the best hit and differing by less than 10% of its identity percentage. The LCA is the lower rank taxon common to most hits, since a small number of hits belonging to other taxa are allowed to add resilience against, for instance, annotation errors. Importantly, our algorithm includes strict cut-off identity values for the various taxonomic ranks. This means that hits must pass a minimum amino acid identity level to be used for assigning to a particular taxonomic rank. These thresholds are 85, 60, 55, 50, 46, 42, and 40% for species, genus, family, order, class, phylum, and superkingdom ranks, respectively (Luo et al., 2014). Hits below these identity levels cannot be used to make assignments to the corresponding rank. For instance, a protein will not be assigned to species level if it has no hits above 85% identity. Moreover, a protein will remain unclassified if it has no hits above 40% identity. Inclusion of these thresholds guarantees that no assignments are performed based on weak, inconclusive hits.

### Functional Assignments

Genes in COGs and KEGG IDs can be annotated using the classical best hit approach or a more sensitive one considering the consistency of all hits (Supplementary Methods in Supplementary File S1). In short, the first hits exceeding an identity threshold for each COG or KEGG are selected. Their bitscores are averaged, and the ORF is assigned to the highest-scoring COG or KEGG whose score exceeds the score of any other by 20%, otherwise the gene remains unannotated. This procedure does not annotate conflicting genes with close similarities to more than one protein family.

### Taxonomic Assignment of Contigs and Disparity Check

The taxonomic assignments of individual genes are used to produce consensus assignments for the contigs. A contig is annotated to the taxon to which most of their genes belong (Supplementary File S1). The required percentage of genes assigned to that taxon can be set by the user, so that it is possible to accommodate missing or incorrect annotations of a few genes, recent HGT events, etc. A disparity score is computed for each contig, indicating how many genes do not concur with the consensus (Supplementary File S1). Contigs with high disparity could be flagged to be excluded from subsequent analyses.

### Coverage and Abundance Estimation for Genes and Contigs

To estimate the abundance of each gene and each contig in each sample, SqueezeMeta relies on mapping of original reads onto the contigs resulting from the assembly. The software Bowtie2 (Langmead and Salzberg, 2012) is used for this task, but we also included Minimap2 (Li, 2018) for mapping long MinION reads. This is followed by Bedtools (Quinlan and Hall, 2010) for extraction of the raw number of reads and bases mapping to each gene and contig. Custom scripts are used to compute the average coverage and normalized RPKM values that provide information on gene and contig abundance.

In sequential mode, SqueezeMeta would stop here. Any of the co-assembly modes allow binning the contigs for delineating genomes.

### Binning

Using the previously obtained contig coverage in different samples, SqueezeMeta uses different binning methods to separate contigs putatively coming from the same organism. Basically, binning algorithms classify contigs coming from the same genomes because their coverages covary along the samples, and their oligonucleotide composition is similar. Currently, Maxbin (Wu et al., 2015) and Metabat2 (Kang et al., 2015) are supported. In addition, SqueezeMeta includes DAS Tool (Sieber et al., 2018) to merge the multiple binning results in just one set.

SqueezeMeta calculates average coverage and RPKM values for the bins in the same way as above, mapping reads to the contigs belonging to the bin.

### Taxonomic Assignment of Bins and Consistency Check

SqueezeMeta generates a consensus taxonomic assignment for the bins in the same way as it did for the contigs. A bin is annotated to the consensus taxon, that is, the taxon to which most of its contigs belong. As previously, a disparity score is computed for each bin, indicating how many of the contigs are discordant with the bin’s consensus taxonomic assignment. This can be used as an initial measure of the bin’s possible contamination.

### Bin Check

The goodness of the bins is estimated using the CheckM software (Parks et al., 2015). In short, CheckM provides indications of a bin’s completeness, contamination and strain heterogeneity by creating a profile of single-copy, conserved genes for the given taxon and evaluating how many of these genes were found (completeness), and how many were single-copy (contamination and strain heterogeneity). SqueezeMeta automates CheckM runs for each bin, using the consensus annotation for the bin as the suggested taxonomic origin.

### Merging of Results

Finally, the system merges all these results and generates several tables: (1) a gene table, with all the information regarding genes (taxonomy, function, contig and bin origin, abundance in samples, and amino acid sequence). (2) A contig table, gathering all data for the contigs (taxonomy, bin affiliation, abundance in samples, and disparity), and (3) A bin table with all information related to the bins (taxonomy, completeness, contamination, abundance in samples, and disparity).

### Database Creation

**FIGURE 2:**
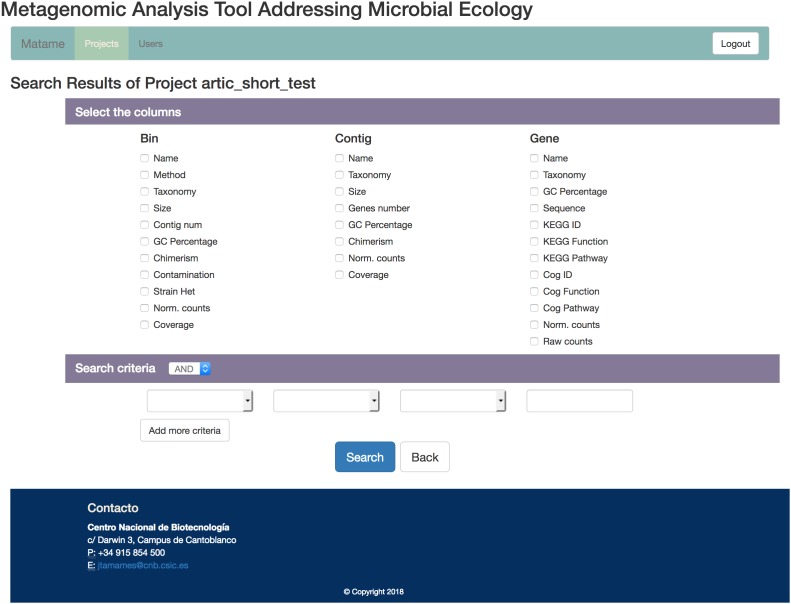
Snapshot of the SqueezeMeta user interface to its database. A flexible and intuitive system for building queries allows interrogating the database with complex questions involving combination of data from different tables.

These three tables and the optional metadata will be used to create a MySQL database for easy inspection of the data arising from the analysis. The database includes a web-based user interface that enables easy creation of queries, so that the user does not need to have any knowledge on database usage to operate it (Figure 2). The interface allows queries on one table (genes, contigs or bins) or combinations of tables, enabling complex questions such as “Retrieve contigs having genes related to trehalose from Bacteroidetes more abundant than 5x coverage in sample X” or “Retrieve antibiotic resistance genes active in one condition but not in another”. The resulting information can be exported to a table.

When combining metagenomes and metatrancriptomes, the latter can be analyzed in a straightforward way by just mapping the cDNA reads against the reference metagenomes. In this way, we can obtain and compare the abundances of the same genes in both the metagenome and the metatranscriptome. However, this will obviate these genes present only in the latter, for instance genes belonging to rare species in the metagenome (therefore unassembled) and that happen to be very active. SqueezeMeta can deal with this situation using the merged mode. Metagenomes and metatranscriptomes are assembled separately and then merged so that contigs can come from DNA from the metagenome, cDNA from the metatranscriptome or both. Normalization of read counts makes it possible to compare presence and expression values within or between different samples.

## Results

**FIGURE 3:**
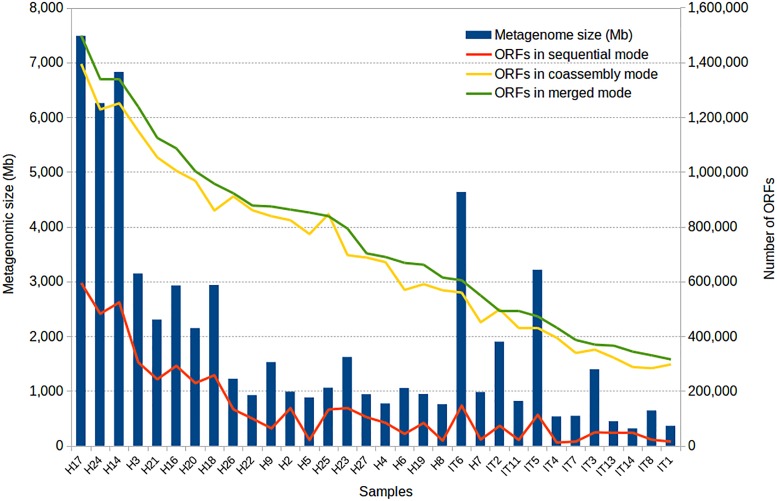
Results of the application of SqueezeMeta to 32 gut metagenomes of Hadza (H) and Italian (IT) subjects. The figure shows the size of the metagenomes and the number of genes obtained by the three modes of analysis.

To illustrate the use of the SqueezeMeta software, we analyzed 32 metagenomic samples corresponding to gut microbiomes of Hadza and Italian subjects (Rampelli et al., 2015), using the three modes of analysis. The total number of reads for all metagenomes is 829.163.742. We used a 64-CPU computer cluster with 756 GB RAM in the National Center for Biotechnology, Madrid, Spain. After discarding contigs below 200 bps, the total number of genes was 4,613,697, 2,401,848, and 2,230,717 for the sequential, merged and co-assembled modes, respectively. Notice that the number of genes is lower in the two latter modes that involve co-assembly since the genes present in more than one metagenome will be counted just once in the co-assembly (they are represented by just one contig product of the co-assembly) but more than once in the individual samples (they are present in one different contig per sample). A more accurate comparison is shown in Figure 3, where a gene in the co-assembly is assumed to be present in a given sample if it can recruit some reads from that sample. As co-assemblies create a much larger reference collection of contigs than individual metagenomes alone, even genes represented by a few reads in a sample can be identified by recruitment, while they will probably fail to assemble in the individual metagenome because of their low abundance. In other words, co-assembly will produce contigs and genes from abundant taxa in one or more samples, that can be used to identify the presence of the same genes in samples in which these original taxa are rare. Therefore, it enables discovering the presence of many more genes in each sample.

The improvement of gene recovery for the smaller samples is also noticeable by the percentage of mapped reads. The individual assembly for small samples achieves barely 35% of read mapping to the assembled metagenome, indicating that most reads could not be used. The small size (and therefore low coverage) of the metagenome prevented these reads from being assembled. When co-assembling these samples with the rest, more than 85% of the reads could then be mapped to the reference metagenome, indicating that co-assembly is able to capture most of the diversity found in these small samples.

Table 2 shows the characteristics of the analysis. Even if the merged mode obtains more contigs and genes than the co-assembly mode, we can see that the number of putatively inconsistent contigs (having genes annotated to different taxa) is lower in the second. Therefore, the co-assembly mode is more accurate than the merged mode, but the latter has the advantage of being able to work with an almost unlimited number of metagenomes because of its lower requirements.

**Table 2:** Statistics on contigs and bins for the three SqueezeMeta modes on Hadza & Italian metagenomes.

	Merged mode	Co-assembly mode	Sequential mode
Number of contigs	893,438	983,350	2,478,560
N50	3900	2357	2854
Average percentage of mapped reads	85.01	89.47	74.47
Contigs with phylum annotation	719,098 (80.4%)	759,903 (77.2%)	1,951,445 (78.7%)
Contigs with disparity > 0	6626 (0.7%)	3772 (0.4%)	7588 (0.3%)
Highly inconsistent contigs (disparity > 0.25)	4496 (0.5%)	2993 (0.3%)	5433 (0.2%)
Number of genes	2,401,848	2,230,717	4,613,697
Genes with COG function	1,098,635 (45.7%)	982,029 (44.0%)	2,164,980 (46.9%)
Genes with KEGG function	835,498 (34.8%)	749,892 (33.6%)	1,683,636 (36.5%)
Total bins	563	423	N/A
Bins > 90% complete	120	115	N/A
Bins > 50% complete	359	192	N/A
High-quality bins (>90% complete, <10% contam)	50	67	N/A
Good quality bins (>75% complete, <10% contam)	82	112	N/A


*Binning statistics refer to MaxBin results.*

Binning results have been analyzed according to the completeness and contamination values provided by CheckM (Table 3). Again, there are differences between the merged and the co-assembly modes, with the first providing more but less complete bins, and the latter giving bins of higher quality. Both modes are capable of obtaining quasi-complete genomes for tens of species, and hundreds of less complete genomes.

**Table 3:** Example of some relevant high-quality bins (>90% completion, <10% contamination) obtained by the co-assembly mode of Hadza & Italian metagenomes.

Taxa	Size (bp)	Completeness	Contamination
o: Clostridiales	3,098,646	99.53%	3.57%
g: Bacteroides	3,521,779	99.45%	0.18%
o: Aeromonadales	2,579,625	99.16%	0.69%
g: Akkermansia	3,031,328	98.94%	4.31%
g: Treponema	2,879,091	98.62%	4.78%
g: Prevotella	3,354,102	97.51%	0.47%
s: *Escherichia coli*	4,710,119	97.49%	6.01%
g: Bifidobacterium	2,266,937	97.40%	3.70%
g: Megasphaera	2,490,127	96.93%	2.88%
s: *Succinatimonas sp.*	2,250,348	96.61%	4.22%
g: Parabacteroides	4,558,677	96.57%	3.18%
s: *Alistipes putredinis*	2,258,860	95.28%	5.77%
s: *Oscillibacter sp.*	1,801,182	95.11%	4.20%
s: *Bacteroides sp.*	3,901,726	95.08%	7.76%


*Taxa are labeled according to their taxonomic rank. g, Genus; o, Order; s, Species.*

**FIGURE 4:**
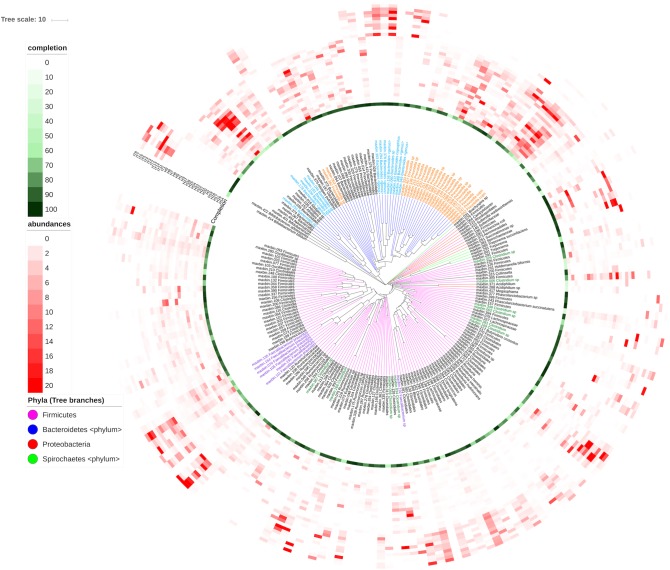
Abundance of bins in the diverse samples. Bins were compared with the CompareM software (https://github.com/dparks1134/CompareM) to estimate their reciprocal similarities. The distances calculated between the bins were used to create a phylogenetic tree illustrating their relationships. The tree is shown in the inner part of the Figure. Branches in the tree corresponding to the four more abundant phyla in the tree (Firmicutes, Bacteriodetes, Proteobacteria, and Spirochaetes) were colored. Bins were named with their id number and original genera, and labels for the most abundant genera were also colored. Outer circles correspond to: the completeness of the bins (green-colored, most internal circle), and the abundance of each bin in each sample (red-colored). Each circle corresponds to a different sample (H, Hadza; I, Italians), and the red color intensities correspond to the bin’s abundance in the sample. The picture was prepared using the iTOL software (https://itol.embl.de).

Figure 4 shows the abundance distribution of bins in samples. Italian subjects reveal a clear distinctive profile that make them cluster together. Bins belonging to the genera *Bacteoides* and *Faecalibacterium* are more abundant in these individuals than in Hadza individuals. The Hadza have increased diversity and fall into different groups corresponding to the presence of diverse species, in accordance with the distinctions found using functional profiles (Rampelli et al., 2015). The microbiota of these individuals contains genera such as *Allistipes* or *Prevotella* not present in the Italian metagenomes. Moreover, Spirochaetes from the genera *Treponema* are only present in Hadza subjects, which are supposedly not associated with pathogenesis. This information is directly retrieved from SqueezeMeta results and offers a revealing view of the genomic composition and differences between the samples. A similar result can be obtained for the functional annotations. The original functions represented in the bins can be used to infer the presence of metabolic pathways using the MinPath algorithm (Ye and Doak, 2009), that defines each pathway as an unstructured gene set and selects the fewest pathways that can account for the genes observed within each bin. The inference of several carbohydrate degradation pathways in the bins can be observed in Supplementary Figure S1.

One of the motivations for the development of SqueezeMeta was making it capable of performing a full metagenomic analysis on a limited computing infrastructure, such as the one that can be expected in the course of *in situ* metagenomic sequencing (Lim et al., 2014; Johnson et al., 2017). We created a setting mode (–lowmem) carefully tailored to run with limited amounts of resources, especially RAM memory. To test this capability, we were able to co-assemble two metagenomic samples from the Hadza metagenomes, composed of 40 million reads amounting to almost 4 GB of DNA sequence. We ran the merged mode of SqueezeMeta using the – low-memory option in a standard laptop computer, using just 8 cores and 16 GB RAM. The run was completed in 10 h, generating 33,660 contigs in 38 bins and 124,065 functionally and taxonomically annotated genes. Using the same settings, we also co-assembled ten MinION metagenomes from the gut microbiome sequencing of head and neck cancer patients^1^, summing 581 MB in less than 4 h. These experiments reveal that SqueezeMeta can be run even with scarce computational resources, and it is suitable for its intended use of *in situ* sequencing where the metagenomes will be moderate in size.

## Discussion

SqueezeMeta is a highly versatile pipeline that enables analyzing a large number of metagenomes or metatranscriptomes in a very straightforward way. All analysis steps are included, starting with assembly, subsequent taxonomic/functional assignment of the resulting genes, abundance estimation and binning to obtain as many genomes as possible in the samples. SqueezeMeta is designed to run in moderately-sized computational infrastructures, relieving the burden of co-assembling tens of metagenomes by using sequential metagenomic assembly and ulterior merging of resulting contigs. The software includes specific software and adjustments to be able to process MinION sequences.

The program includes several verifications on the results, such as the detection of possible inconsistent contigs and bins, and estimation of the latter’s completion using the checkM software. Finally, results can easily be inspected and managed since SqueezeMeta includes a built-in MySQL database that can be queried via a web-based interface, allowing the creation of complex queries in a very simple way.

One of the most remarkable features of this software is its capability to operate in limited computing infrastructure. We were able to analyze several metagenomes in a few hours using a virtual machine with just 16 GB RAM. Therefore, SqueezeMeta is apt to be used in scenarios in which computing resources are limited, such as remote locations in the course of metagenomic sampling campaigns. Also, it does not require the availability of any Internet connection. Obviously, complex, sizeable metagenomes cannot be analyzed with these limited resources. However, the intended use of *in situ* sequencing will likely produce a moderate and manageable data size.

SqueezeMeta will be further expanded by the creation of new tools allowing in-depth analyses of the functions and metabolic pathways represented in the samples.

## Author Contributions

JT conceived and designed the tool and wrote the manuscript. JT and FP-S created the software and performed all necessary testing and read and approved the manuscript.

## Conflict of Interest Statement

The authors declare that the research was conducted in the absence of any commercial or financial relationships that could be construed as a potential conflict of interest.

## Footnotes

1 https://www.ncbi.nlm.nih.gov/bioproject/PRJNA493153

## Supplementary Material

FIGURE S1 The presence of several carbohydrate degradation pathways in the bins. The outer circles indicate the percentage of genes from a pathway present in each of the bins. According to that gene profile, MinPath estimates whether or not the pathway is present. Only pathways inferred to be present are colored. As in Figure 4, the bins tree is performed from a distance matrix of the orthologous genes’ amino acid identity, using the compareM software (https://github.com/dparks1134/CompareM). The four most abundant phyla are colored (branches in the tree), as well as the most abundant genera (bin labels). The picture was elaborated using the iTOL software (https://itol.embl.de).

FILE S1 Description of novel algorithms implemented in SqueezeMeta.

## References

[B1] Abubucker S. Segata N. Goll J. Schubert A. M. Izard J. Cantarel B. L. (2012). Metabolic reconstruction for metagenomic data and its application to the human microbiome. *PLoS Comput. Biol.* 8:2358. 10.1371/journal.pcbi.1002358 22719234 PMC3374609

[B2] Arumugam M. Harrington E. D. Foerstner K. U. Raes J. Bork P. (2010). SmashCommunity: a metagenomic annotation and analysis tool. *Bioinformatics* 26 2977–2978. 10.1093/bioinformatics/btq536 20959381

[B3] Bankevich A. Nurk S. Antipov D. Gurevich A. A. Dvorkin M. Kulikov A. S. (2012). SPAdes: a new genome assembly algorithm and its applications to single-cell sequencing. *J. Comput. Biol.* 19 455–477. 10.1089/cmb.2012.0021 22506599 PMC3342519

[B4] Bolger A. M. Lohse M. Usadel B. (2014). Trimmomatic: a flexible trimmer for Illumina sequence data. *Bioinformatics* 30 2114–2120. 10.1093/bioinformatics/btu170 24695404 PMC4103590

[B5] Buchfink B. Xie C. Huson D. H. (2015). Fast and sensitive protein alignment using DIAMOND. *Nat. Methods* 12 59–60. 10.1038/nmeth.3176 25402007

[B6] Carr R. Borenstein E. (2014). Comparative analysis of functional metagenomic annotation and the mappability of short reads. *PLoS One* 9:e105776. 10.1371/journal.pone.0105776 25148512 PMC4141809

[B7] Deamer D. Akeson M. Branton D. (2016). Three decades of nanopore sequencing. *Nat. Biotechnol.* 34 518–524. 10.1038/nbt.3423 27153285 PMC6733523

[B8] Eddy S. R. (2009). A new generation of homology search tools based on probabilistic inference. *Genome Inform.* 23 205–211. 10.1142/9781848165632_0019 20180275

[B9] Eren A. M. Esen C. Quince C. Vineis J. H. Morrison H. G. Sogin M. L. (2015). Anvi’o: an advanced analysis and visualization platform for ‘omics data. *PeerJ* 3 1–29. 10.7717/peerj.1319 26500826 PMC4614810

[B10] Finn R. D. Bateman A. Clements J. Coggill P. Eberhardt R. Y. Eddy S. R. (2014). Pfam: the protein families database. *Nucleic Acids Res.* 42 D222–D230. 24288371 10.1093/nar/gkt1223 PMC3965110

[B11] Fu L. Niu B. Zhu Z. Wu S. Li W. (2012). CD-HIT: accelerated for clustering the next-generation sequencing data. *Bioinformatics* 28 3150–3152. 10.1093/bioinformatics/bts565 23060610 PMC3516142

[B12] Glass E. M. Meyer F. (2011). “The metagenomics RAST server: a public resource for the automatic phylogenetic and functional analysis of metagenomes,” in *Handbook of Molecular Microbial Ecology I: Metagenomics and Complementary Approaches*, (Hoboken, NJ: Wiley Blackwell), 325–331. 10.1002/9781118010518.ch37 PMC2563014 18803844

[B13] Hitch T. C. A. Creevey C. J. (2018). Spherical: an iterative workflow for assembling metagenomic datasets. *BMC Bioinformatics* 19:2. 10.1186/s12859-018-2028-2 29361904 PMC5781261

[B14] Huerta-Cepas J. Szklarczyk D. Forslund K. Cook H. Heller D. Walter M. C. (2016). EGGNOG 4.5: a hierarchical orthology framework with improved functional annotations for eukaryotic, prokaryotic and viral sequences. *Nucleic Acids Res.* 44 D286–D293. 10.1093/nar/gkv1248 26582926 PMC4702882

[B15] Hyatt D. Chen G. L. LoCascio P. F. Land M. L. Larimer F. W. Hauser L. J. (2010). Prodigal: prokaryotic gene recognition and translation initiation site identification. *BMC Bioinformatics* 11:119. 10.1186/1471-2105-11-119 20211023 PMC2848648

[B16] Johnson S. S. Zaikova E. Goerlitz D. S. Bai Y. Tighe S. W. (2017). Real-time DNA sequencing in the antarctic dry valleys using the Oxford nanopore sequencer. *J. Biomol. Tech.* 28 2–7. 10.7171/jbt.17-2801-009 28337073 PMC5362188

[B17] Kanehisa M. Goto S. (2000). KEGG: kyoto encyclopaedia of genes and genomes. *Nucleic Acids Res.* 28 27–30. 10.1093/nar/28.1.27 10592173 PMC102409

[B18] Kang D. D. Froula J. Egan R. Wang Z. (2015). MetaBAT, an efficient tool for accurately reconstructing single genomes from complex microbial communities. *PeerJ* 3:e1165. 10.7717/peerj.1165 26336640 PMC4556158

[B19] Kim J. Kim M. S. Koh A. Y. Xie Y. Zhan X. (2016). FMAP: functional mapping and analysis pipeline for metagenomics and metatranscriptomics studies. *BMC Bioinformatics* 17:420. 10.1186/s12859-016-1278-0 27724866 PMC5057277

[B20] Koren S. Walenz B. P. Berlin K. Miller J. R. Bergman N. H. Phillippy A. M. (2017). Canu: scalable and accurate long-read assembly via adaptive κ-mer weighting and repeat separation. *Genome Res.* 27 722–736. 10.1101/gr.215087.116 28298431 PMC5411767

[B21] Langmead B. Salzberg S. L. (2012). Fast gapped-read alignment with Bowtie 2. *Nat. Methods* 9 357–359. 10.1038/nmeth.1923 22388286 PMC3322381

[B22] Li D. Liu C. M. Luo R. Sadakane K. Lam T. W. (2015). MEGAHIT: an ultra-fast single-node solution for large and complex metagenomics assembly via succinct de Bruijn graph. *Bioinformatics* 31 1674–1676. 10.1093/bioinformatics/btv033 25609793

[B23] Li H. (2018). Minimap2: pairwise alignment for nucleotide sequences. *Bioinformatics* 34 3094–3100. 10.1093/bioinformatics/bty191 29750242 PMC6137996

[B24] Li W. (2009). Analysis and comparison of very large metagenomes with fast clustering and functional annotation. *BMC Bioinformatics* 10:359. 10.1186/1471-2105-10-359 19863816 PMC2774329

[B25] Lim Y. W. Cuevas D. A. Silva G. G. Z. Aguinaldo K. Dinsdale E. A. Haas A. F. (2014). Sequencing at sea: challenges and experiences in Ion Torrent PGM sequencing during the 2013 Southern Line Islands Research Expedition. *PeerJ* 2:e520. 10.7717/peerj.520 25177534 PMC4145072

[B26] Luo C. Rodriguez-R L. M. Konstantinidis K. T. (2014). MyTaxa: an advanced taxonomic classifier for genomic and metagenomic sequences. *Nucleic Acids Res.* 42:e73. 10.1093/nar/gku169 24589583 PMC4005636

[B27] Meyer F. Paarmann D. D’Souza M. Olson R. Glass E. M. Kubal M. (2008). The metagenomics RAST server—a public resource for the automatic phylo- genetic and functional analysis of metagenomes. *BMC Bioinformatics* 9:386. 10.1186/1471-2105-9-386 18803844 PMC2563014

[B28] Narayanasamy S. Jarosz Y. Muller E. E. L. Heintz-Buschart A. Herold M. Kaysen A. (2016). IMP: a pipeline for reproducible reference-independent integrated metagenomic and metatranscriptomic analyses. *Genome Biol.* 17:260. 10.1186/s13059-016-1116-8 27986083 PMC5159968

[B29] Parks D. H. Imelfort M. Skennerton C. T. Hugenholtz P. Tyson G. W. (2015). CheckM: assessing the quality of microbial genomes recovered from isolates, single cells, and metagenomes. *Genome Res.* 25 1043–1055. 10.1101/gr.186072.114 25977477 PMC4484387

[B30] Pignatelli M. Aparicio G. Blanquer I. Hernández V. Moya A. Tamames J. (2008). Metagenomics reveals our incomplete knowledge of global diversity. *Bioinformatics* 24 2124–2125. 10.1093/bioinformatics/btn355 18625611 PMC2530889

[B31] Quick J. Ashton P. Calus S. Chatt C. Gossain S. Hawker J. (2015). Rapid draft sequencing and real-time nanopore sequencing in a hospital outbreak of *Salmonella*. *Genome Biol.* 16:114. 10.1186/s13059-015-0677-2 26025440 PMC4702336

[B32] Quick J. Loman N. J. Duraffour S. Simpson J. T. Severi E. Cowley L. (2016). Real-time, portable genome sequencing for Ebola surveillance. *Nature* 530 228–232. 10.1038/nature16996 26840485 PMC4817224

[B33] Quinlan A. R. Hall I. M. (2010). BEDTools: a flexible suite of utilities for comparing genomic features. *Bioinformatics* 26 841–842. 10.1093/bioinformatics/btq033 20110278 PMC2832824

[B34] Rampelli S. Schnorr S. L. Consolandi C. Turroni S. Severgnini M. Peano C. (2015). Metagenome sequencing of the hadza hunter-gatherer gut microbiota. *Curr. Biol.* 25 1682–1693. 10.1016/j.cub.2015.04.055 25981789

[B35] Schmieder R. Edwards R. (2011). Quality control and preprocessing of metagenomic datasets. *Bioinformatics* 27 863–864. 10.1093/bioinformatics/btr026 21278185 PMC3051327

[B36] Seemann T. (2014). Prokka: rapid prokaryotic genome annotation. *Bioinformatics* 30 2068–2069. 10.1093/bioinformatics/btu153 24642063

[B37] Sieber C. M. K. Probst A. J. Sharrar A. Thomas B. C. Hess M. Tringe S. G. (2018). Recovery of genomes from metagenomes via a dereplication, aggregation and scoring strategy. *Nat. Microbiol.* 3 836–843. 10.1038/s41564-018-0171-1 29807988 PMC6786971

[B38] Tamames J. Puente-Sanchez F. (2018). SqueezeM, a fully automatic metagenomic analysis pipeline from reads to bins. *bioRxiv* [Preprint]. 10.1101/347559 PMC6353838 30733714

[B39] Treangen T. J. Sommer D. D. Angly F. E. Koren S. Pop M. (2011). Next generation sequence assembly with AMOS. *Curr. Protoc.* 33 11.8.1–11.8.18. 10.1002/0471250953.bi1108s33 21400694 PMC3072823

[B40] Tully B. J. Graham E. D. Heidelberg J. F. (2018). The reconstruction of 2,631 draft metagenome-assembled genomes from the global oceans. *Sci. Data* 5:170203. 10.1038/sdata.2017.203 29337314 PMC5769542

[B41] Uritskiy G. V. DiRuggiero J. Taylor J. (2018). MetaWRAP - a flexible pipeline for genome-resolved metagenomic data analysis. *Microbiome* 6:158. 10.1186/s40168-018-0541-1 30219103 PMC6138922

[B42] Wang Q. Garrity G. M. Tiedje J. M. Cole J. R. (2007). Naïve Bayesian classifier for rapid assignment of rRNA sequences into the new bacterial taxonomy. *Appl. Environ. Microbiol.* 73 5261–5267. 10.1128/AEM.00062-07 17586664 PMC1950982

[B43] Westreich S. T. Treiber M. L. Mills D. A. Korf I. Lemay D. G. (2018). SAMSA2: a standalone metatranscriptome analysis pipeline. *BMC Bioinformatics* 19:175. 10.1186/s12859-018-2189-z 29783945 PMC5963165

[B44] Wommack K. E. Bhavsar J. Ravel J. (2008). Metagenomics: read length matters. *Appl. Environ. Microbiol.* 74 1453–1463. 10.1128/AEM.02181-07 18192407 PMC2258652

[B45] Wu Y. W. Simmons B. A. Singer S. W. (2015). MaxBin 2.0: an automated binning algorithm to recover genomes from multiple metagenomic datasets. *Bioinformatics* 32 605–607. 10.1093/bioinformatics/btv638 26515820

[B46] Ye Y. Doak T. G. (2009). A parsimony approach to biological pathway reconstruction/inference for genomes and metagenomes. *PLoS Comput. Biol.* 5:465. 10.1371/journal.pcbi.1000465 19680427 PMC2714467

