# Supplementary figures and images for: SqueezeMeta, A Highly Portable, Fully Automatic Metagenomic Analysis Pipeline
Source: Front Microbiol. 2019 Jan 24;9:3349. doi: 10.3389/fmicb.2018.03349 (PMC6353838; doi:10.3389/fmicb.2018.03349)

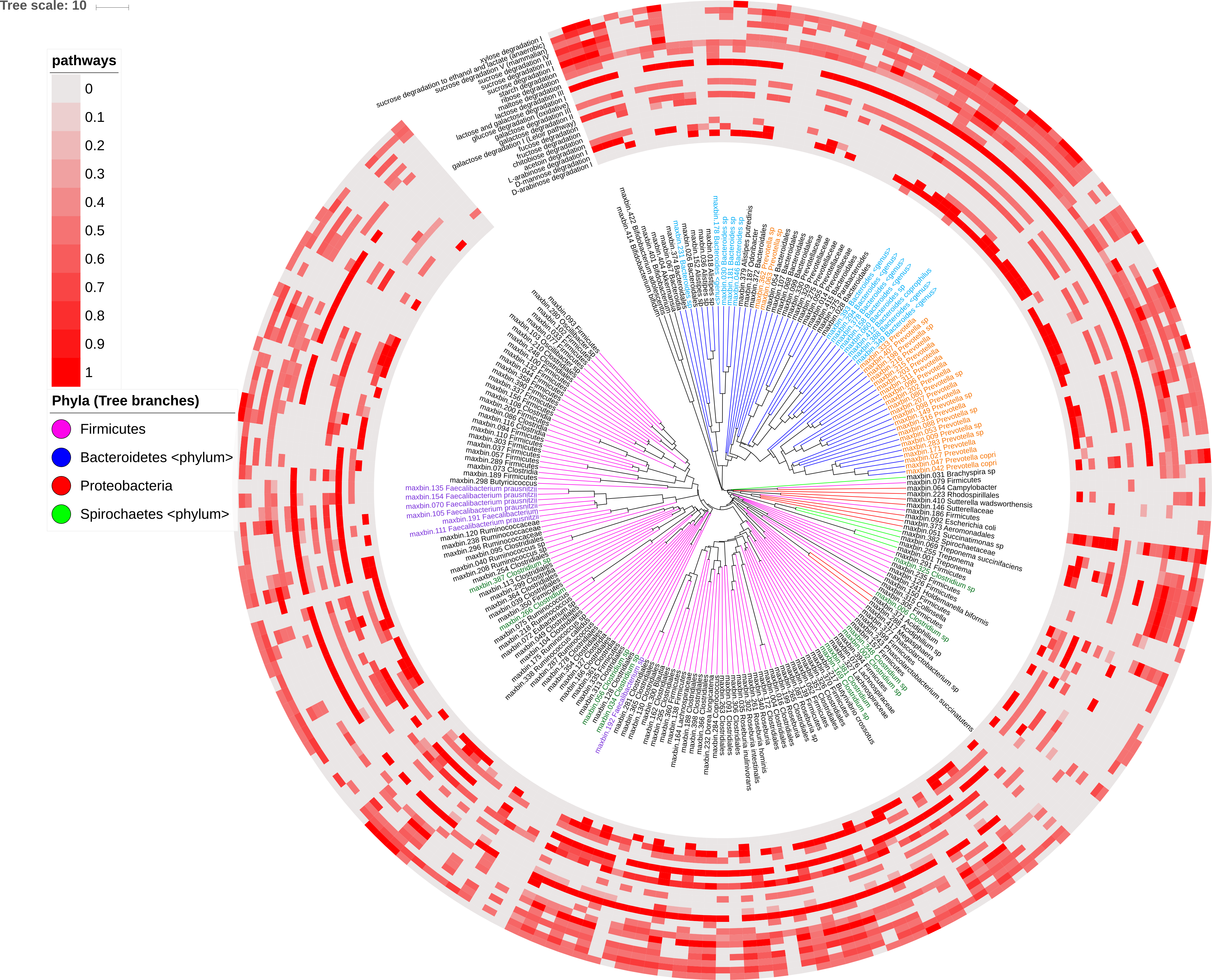

Supplement: FIGURE S1 — The presence of several carbohydrate degradation pathways in the bins. The outer circles indicate the percentage of genes from a pathway present in each of the bins. According to that gene profile, MinPath estimates whether or not the pathway is present. Only pathways inferred to be present are colored. As in Figure 4, the bins tree is performed from a distance matrix of the orthologous genes’ amino acid identity, using the compareM software (https://github.com/dparks1134/CompareM). The four most abundant phyla are colored (branches in the tree), as well as the most abundant genera (bin labels). The picture was elaborated using the iTOL software (https://itol.embl.de). [file Image_1.PNG]
