# Supplementary material for: SqueezeMeta, A Highly Portable, Fully Automatic Metagenomic Analysis Pipeline
Source: Front Microbiol. 2019 Jan 24;9:3349. doi: 10.3389/fmicb.2018.03349 (PMC6353838; doi:10.3389/fmicb.2018.03349)
Supplement: FILE S1 — Description of novel algorithms implemented in SqueezeMeta. [file Table_1.DOCX]

SqueezeM, a fully automatic metagenomic analysis pipeline from reads to bins

Javier Tamames*, Fernando Puente-Sánchez

Departamento de Biología de Sistemas, Centro Nacional de Biotecnología, CSIC, C/Darwin 3, 28045 Cantoblanco, Madrid, Spain

**Description of squeezeM algorithms LCA, consensus calculation, and chimerism calculation**

**The LCA algorithm**

We use a Last Common Ancestor (LCA) algorithm to assign taxa to genes.

For the aminoacid sequence of each gene, diamond (blastp) homology searches are done against the GenBank nr database (updated weekly). A e-value cutoff of 1e-03 is set by default. The best hit is obtained, and then we select a range of hits (valid hits) having at least 80% of the bitscore of the best hit and differing in less than 10% identity also with the best hit (these values can be set). The LCA of all these hits is obtained, that is, the taxon common to all hits. This LCA can be found at diverse taxonomic ranks (from phylum to species). We allow some flexibility in the definition of LCA: a small number of hits belonging to other taxa than the LCA can be allowed. In this way, we deal with putative transfer events, or incorrect annotations in the database. This value is by default 10% of the total number of valid hits, but can be set by the user. Also, the minimum number of hits to the LCA taxa can be set.

An example is shown in the next table

| GenID | Hit ID | Hit tax | Identity | e-value |
| --- | --- | --- | --- | --- |
| Gen1 | Hit1 | Genus:Polaribacter  Family: Flavobacteriaceae  Order:Flavobacteriales | 75.2 | 1e-94 |
| Gen1 | Hit2 | Genus:Polaribacter  Family: Flavobacteriaceae  Order:Flavobacteriales | 71.3 | 6e-88 |
| Gen1 | Hit3 | Family: Flavobacteriaceae  Order:Flavobacteriales | 70.4 | 2e-87 |
| Gen1 | Hit4 | Genus:Algibacter  Family: Flavobacteriaceae  Order:Flavobacteriales | 68.0 | 2e-83 |
| Gen1 | Hit5 | Genus:Rhodospirillum  Family: Rhodospirillaceae  Order:Rhodospirillales | 60.2 | 6e-68 |

In this case, the four first hits are the valid ones. Hit 5 does not make the identity and e-value thresholds. The LCA for the four valid hits is Family: Flavobacteriaceae, that would be the reported result.

Our LCA algorithm includes strict cut-off identity values for different taxonomic ranks, according to Luo et al, Nucleic Acids Research 2014, 42, e73. This means that hits must pass a minimum (aminoacid) identity level in order to be used for assigning particular taxonomic ranks. These thresholds are 85, 60, 55, 50, 46, 42 and 40% for species, genus, family, order, class, phylum and superkingdom ranks, respectively. Hits below these levels cannot be used to make assignments for the corresponding rank. For instance, a protein will not be assigned to species level if it has no hits above 85% identity. Also, a protein will remain unclassified if it has no hits above 40% identity. The inclusion of these thresholds guarantees that no assignments are done based on weak, inconclusive hits.

**Functional assignment: Best hit/Best average**

The functional assignment of ORFs against eggNOG or KEGG databases can be done using the best hit approach: The annotated function will be the one of the best hit against these databases, if it exceeds a given identity threshold (default: 50%).

A more accurate mode if the best average approach: For a particular ORF, The first n hits (currently n=3) for each COG or KEGG are selected (exceeding the same identity threshold above). Then their bitscores are averaged, and the ORF is assigned to the higher-scoring COG or KEGG whose score exceeds by 20% the score of any other COG/KEGG. Otherwise the ORF remains unannotated. This procedure does not annotate conflicting genes with close similarities to more than one protein family.

**Contig (or bin) consensus algorithm**

The consensus algorithm attempts to obtain a consensus taxonomic annotation for the contigs according to the annotations of each of its genes. The consensus taxon if the one fulfilling:

-50% of the genes of the contig belong to (are annotated to) this taxon, and

-70% of the annotated genes belong to (are annotated to) this taxon.

Notice that the first criterion refers to all genes in the contig, regardless if they have been annotated or nor, while the second refers exclusively to annotated genes.

As the assignment can be done at different taxonomic ranks, the consensus is the deepest taxon fulfilling the criteria above.

For instance, consider the following example for a contig with 6 genes:

Gen1: k_Bacteria;p_Proteobacteria;c_Gamma-Proteobacteria;o_Enterobacteriales;f_ Enterobacteriaceae;g_Escherichia;s_Escherichia coli

Gen2: k_Bacteria;p_Proteobacteria;c_Gamma-Proteobacteria;o_Enterobacteriales;f_ Enterobacteriaceae;g_Escherichia

Gen3: k_Bacteria;p_Proteobacteria;c_Gamma-Proteobacteria;o_Enterobacteriales;f_ Enterobacteriaceae;g_Escherichia

Gen4: k_Bacteria;p_Proteobacteria;c_Gamma-Proteobacteria;o_Enterobacteriales;f_ Enterobacteriaceae

Gen5: No hits

Gen6: k_Bacteria;p_Firmicutes

In this case, the contig will be assigned to k_Bacteria;p_Proteobacteria;c_Gamma-Proteobacteria;o_ Enterobacteriales;f_ Enterobacteriaceae, which is the deepest taxon fulfilling 50% of all the genes belonging to that taxon (4/6=66%), and having 70% of the annotated genes (4/5=80%). It was not possible to annotate the contig to genus Escherichia since just 3/5=60% of the annotated genes belong to it.

For annotating the consensus of bins, the procedure is the same, just using the annotations for the contigs belonging to the bin instead.

**Chimerism calculation**

Notice that in the example above, the end part of the contig seems to depart from the common taxonomic origin of the rest. This is an indication of a potential chimerism produced in the assembly (although it can be die to other causes, such as a recent LCA transfer or a misannotation for the gene). The chimerism index attempts to measure this effect, so that the contigs can be flagged accordingly (for instance, we could decide not trusting highly chimeric contigs).

Chimerism index is calculated for the taxonomic rank assigned by consensus (in the previous example, family). We compare the assignments at that level for every pair of genes in the contig, and count the number of agreements and disagreements. If one of the taxa has no annotation at that level, is not counted for agreement but it is counted for disagreements if previous ranks do not coincide. That is:

Gen1-Gen2: Agree

Gen1-Gen3: Agree

Gen1-Gen4: Agree

Gen1-Gen5: Unknown

Gen1-Gen6: Disagree (at phylum level)

Gen2-Gen3: Agree

Gen2-Gen4: Agree

Gen2-Gen5: Unknown

Gen2-Gen6: Disagree (at phylum level)

Gen3-Gen4: Agree

Gen3-Gen5: Unknown

Gen3-Gen6: Disagree (at phylum level)

Gen4-Gen5: Unknown

Gen4-Gen6: Disagree (at phylum level)

Gen5-Gen6: Unknown

Chimerism index is the ratio between the number of disagreements and the total number of comparisons, in this case 4/15=0.26

For calculating the chimerism of bins, the procedure is the same, just using the annotations for the contigs belonging to the bin instead.
